# Optimum plant density for crowding stress tolerant processing sweet corn

**DOI:** 10.1371/journal.pone.0223107

**Published:** 2019-09-26

**Authors:** Daljeet S. Dhaliwal, Martin M. Williams

**Affiliations:** 1 Department of Crop Sciences, University of Illinois, Urbana, Illinois, United States of America; 2 Global Change and Photosynthesis Research Unit, USDA-ARS, Urbana, Illinois, United States of America; Instituto Agricultura Sostenible, SPAIN

## Abstract

Globally, gains in sweet corn [*Zea mays* L.var. *rugosa* (or *saccharata*)] are a fraction of the yield advances made in field corn (*Zea mays* L.) in the last half-century. Grain yield improvement of field corn is associated with increased tolerance to higher plant densities (i.e., crowding stress). Processing sweet corn hybrids that tolerate crowding stress have been identified; however, such hybrids appear to be under-planted in the processing sweet corn. Using crowding stress tolerant (CST) hybrids, the objectives of this study were to: (1) identify optimum plant densities for a range of growing conditions; (2) quantify gaps in production between current and optimum plant densities; and (3) enumerate changes in yield and ear traits when shifting from current to optimum plant densities. Using a CST shrunken-2 (*sh2*) processing sweet corn hybrid, on-farm plant density trials were conducted in thirty fields across the states of Illinois, Minnesota and Wisconsin, from 2013 to 2017 in order to capture a wide variety of growing conditions. Linear mixed-effects models were used to identify the optimum plant density corresponding to maximum ear mass (Mt ha^-1^), case production (cases ha^-1^), and profitability to the processor ($ ha^-1^). Kernel moisture, indicative of plant development, was unaffected by plant density. Ear traits, such as ear number and ear mass per plant, average ear length, and filled ear length declined linearly with increasing plant density. Nonetheless, there was a large economic benefit to the grower and processor by shifting to higher plant densities in most environments. This research shows that increasing plant densities of CST hybrids from current (58,475 plants ha^-1^) to optimum (73,075 plants ha^-1^) could improve processing sweet corn green ear yield and processor profitability on average of 1.13 Mt ha^-1^ and $525 ha^-1^, respectively.

## Introduction

Over the last 50 years, field corn (*Zea mays L*.) has demonstrated drastic improvements in grain yield, with the United States (U.S.) national average yields increasing from 5 Mt ha^-1^ in 1966 to 11 Mt ha^-1^ in 2016 [1]. Advancements in field corn yield can be attributed to both availability of genetically improved hybrids and adoption of superior agronomic management practices [2]. Furthermore, genetic yield improvements were primarily due to increased stress tolerance [3]. Meanwhile, individual plant yield potential of modern hybrids does not differ substantially from that of old hybrids [4, 5]. Previous studies have reported that gains observed in grain yield are plant density dependent [2, 5, 6].

Tolerance to intense intraspecific competition for available resources has improved more than any other environmental stress tolerance over the past ~50 years [6, 7]. This can be attributed to the shift in hybrid evaluation philosophy in the early 1980s in North America; where instead of emphasizing relatively high precision per location at a few locations, breeders tested hybrid response at many locations, including environments with crowding, nutrient, and water stresses [8]. Such selection criteria, with more reliance on yield stability as opposed to yield increase, is considered responsible for increased stress tolerance in modern hybrids [2, 6].

Unlike field corn, sweet corn has failed to realize any significant yield improvements, where it is grown in several regions of the Americas, Europe, Middle East, Africa, Asia, and Australasia. For instance, processing sweet corn has shown only a 16 percent yield gain compared to 31 percent grain yield gains in field corn over the last two decades (1998–2017) in the U.S. [1]. Genetic improvements of sweet corn have primarily focused on manipulation of different endosperm mutants, specifically shrunken-2 (*sh2*), brittle1 (*bt*), sugary 1 (*su1*), and sugary enhancer 1 (*se*) to develop better tasting and longer shelf-life products [9]. Also, there is evidence of increased host plant resistance to some of the common plant diseases (common rust, maize dwarf mosaic, northern corn leaf blight) prevalent on sweet corn in North America [10]. However, there has been limited investigation into sweet corn plant density relationships with yield and yield components.

Recent research shows that widely used processing sweet corn hybrids differ significantly in crowding stress tolerance (CST) and yield potential [11]. [12] reported two categories of traits, namely photosynthetic capacity and source-sink relationships associated with crowding stress tolerance in processing sweet corn. Genes involved in photosynthesis, glycolysis, cell wall development, carbohydrate/nitrogen metabolic processes, and chromatin as well as transcription regulation processes, are possible mechanisms behind CST in processing sweet corn [13]. Crowding stress tolerant maize hybrids, when planted at their optimum plant density, out-perform hybrids with poor CST [11, 14].

Crowding stress tolerance is a heritable trait in sweet corn [15]. Even though certain processing sweet corn hybrids have above-average CST, field surveys report on plant density has changed little the last two decades, averaging 56,000 plants ha^-1^ in the U.S. [16]. Previous study documented a wide range (48,100 to 70,200 plants ha^-1^) in plant density for maximum green ear yield in six commercial processing sweet corn hybrids [16]. Crowding stress tolerant sweet corn could be grown at higher plant densities than current densities. Consequently, the production gap—the difference in performance between using current and optimum plant densities—can be narrowed.

There is extensive literature on field corn plant density interactions with yield and yield components. In contrast, the inference space of the few studies on plant density and yield relationships in sweet corn is limited by a narrow range of environments in which field trials were conducted (e.g. up to 3 years at one location). An experimental approach that captures greater diversity in the conditions in which the crop is grown, including environments and management systems, would provide more valuable insight. Moreover, existing literature mostly investigates optimum plant density in fresh-market sweet corn [17, 18], which does not apply to the unique hybrids and yield parameters of processing sweet corn [16].

The goal of this research was to determine the extent to which CST could improve yield of sweet corn grown for processing. Using CST hybrids, the objectives of this study were to: (1) identify optimum plant densities for a range of growing conditions; (2) quantify production gaps between current and optimum plant densities; and (3) based on optimizing gross profit margin, enumerate changes in yield and ear traits when shifting from current to optimum plant densities.

## Materials and methods

To address objectives, the general experimental approach involved growing CST hybrids over a range of plant densities under variable environments where processing sweet corn is grown. This required a collaborative effort among the authors, two vegetable processors, and their contract growers. A common research protocol was implemented on-farm, either nested in contract fields or at a university research farm. All aspects of crop management reflected the realities of processing sweet corn production in each production area. A detailed description of the experimental approach is provided below.

### Germplasm

Previous research [11] on 26 *sh2* endosperm type processing sweet corn hybrids documented large variability in crowding stress response. From the hybrids, the two most CST hybrids were selected for this research, specifically ‘DMC 21–84’, a hybrid developed by Del Monte, and ‘GG 641’, a hybrid developed by General Mills. Both hybrids were grown widely in the region, providing numerous contract growers and individual fields from which to select study sites. Early in the project, the processor of GG 641 withdrew from the project. Given the limited availability of access to fields grown with known CST hybrids, and to avoid confounding plant density response with genetic background, DMC 21–84 was the single hybrid used in the project. It is noteworthy that DMC 21–84 is widely grown in the states of Illinois, Minnesota and Wisconsin since 2005 (Dhaliwal and Williams, unpublished data).

### Description of sites

The study was conducted at 30 site-years (hereafter called ‘fields’), located in areas of high strategic importance for sweet corn production in Illinois, Minnesota and Wisconsin, from 2013 to 2017 (Table 1). Soil texture varied from clay loam, silty loam to sand. Soils greater than 50 percent sand were sprinkler irrigated, whereas other soils were rainfed. Planting date ranged from April 24 to June 19. As such, harvest ranged from July 20 to September 26. Sweet corn was grown in rotation with other summer annual crops and conventional tillage practices were used in all fields. With the exception of harvest, trials were maintained such that crop management practices (i.e., irrigation, nutrient management, disease and pest control, weed control) were not differentiated between the trial and the field in which the trial was nested. Fields were selected from four production areas; geographically separate areas in which processors currently make different crop management decisions. Production areas were named Illinois-irrigated, Illinois-rainfed, Minnesota-rainfed and Wisconsin-irrigated to indicate the state and water supply of each field.

**Table 1:** Site characterization of fields employed in on-farm plant density trials.

Year	State	County	Name	Soil texture	Water supply	Planting date	Harvest date
2013	IL	LaSalle	MD_Y13	Silt loam	Rainfed	19-Jun	6-Sep
2014	IL	Champaign	FF_Y14	Silt loam	Rainfed	27-May	11-Aug
2014	IL	Champaign	VC_Y14	Silt loam	Rainfed	27-May	13-Aug
2014	IL	DeKalb	TYLR1_Y14	Silt loam	Rainfed	6-Jun	29-Aug
2014	IL	DeKalb	TYLR2_Y14	Silt loam	Rainfed	6-Jun	29-Aug
2014	IL	LaSalle	UTI_Y14	Silt loam	Rainfed	14-Jun	5-Sep
2014	WI	Portage	OKR_Y14	Loamy sand	Irrigated	19-Jun	18-Sep
2014	WI	Portage	PMT_Y14	Muck sand	Irrigated	5-Jun	9-Sep
2014	WI	Portage	WYN_Y14	Loamy sand	Irrigated	23-May	25-Aug
2015	IL	Champaign	FF_Y15	Silt loam	Rainfed	22-May	5-Aug
2015	IL	Champaign	VC_Y15	Silt loam	Rainfed	22-May	6-Aug
2015	IL	Mason	HV_Y15	Sandy loam	Irrigated	29-Apr	20-Jul
2015	MN	Brown	HOFF_Y15	Clay loam	Rainfed	10-Jun	4-Sep
2015	MN	Redwood	HOFS_Y15	Clay loam	Rainfed	10-Jun	4-Sep
2015	WI	Portage	PMT_Y15	Loamy sand	Irrigated	2-Jun	3-Sep
2015	WI	Portage	WY_Y15	Loamy sand	Irrigated	13-May	20-Aug
2015	WI	Waushara	MRT_FY15	Loamy sand	Irrigated	16-Jun	15-Sep
2016	IL	Champaign	FF_Y16	Silt loam	Rainfed	16-May	1-Aug
2016	IL	Champaign	VC_Y16	Silt loam	Rainfed	16-May	1-Aug
2016	IL	Mason	HV_Y16	Sandy loam	Irrigated	20-Apr	22-Jul
2016	MN	Brown	HOFS_Y16	Clay loam	Rainfed	13-Jun	31-Aug
2016	WI	Adams	AIR_Y16	Loamy sand	Irrigated	1-Jun	23-Aug
2016	WI	Portage	P15_Y16	Muck sand	Irrigated	8-Jun	6-Sep
2016	WI	Portage	TIMM_Y16	Loamy sand	Irrigated	19-Jun	14-Sep
2017	IL	Champaign	M11_Y17	Silt loam	Rainfed	24-Apr	28-Jul
2017	IL	Champaign	VC_Y17	Silt loam	Irrigated	16-May	7-Aug
2017	MN	Brown	HOFS1_Y17	Clay loam	Rainfed	10-Jun	7-Sep
2017	MN	Brown	HOFS2_Y17	Clay loam	Rainfed	11-Jun	7-Sep
2017	WI	Portage	PL1_Y17	Sand	Irrigated	30-May	31-Aug
2017	WI	Portage	PL2_Y17	Loamy sand	Irrigated	23-Jun	26-Sep

### Experimental design

All trials were laid out as a randomized complete block design with two replicates. Treatments consisted of ten target plant densities, i.e., 42,000, 49,000, 57,000, 64,000, 72,000, 79,000, 86,000, 94,000, 101,000 and 109,000 plants ha^-1^. Plot size varied by field according to available space and size of planting equipment; however, all sweet corn was grown on a 76 cm row spacing.

### Data collection and analysis

#### Harvest data

Sweet corn is harvested at the ‘milk’ stage (R3). For *sh2* hybrids, ideal kernel moisture at harvest is 76 percent. In this study, harvest date of contract fields was decided by the processor. Trials were harvested promptly before machine harvest of the field in which trials were nested, or in the case of the university research farm, when kernel moisture was near 76 percent. The harvest area was within two interior rows (i.e., to avoid border effects) from each plot over a length of 6.1 meter. Green ears measuring ≥ 4.5 cm in diameter (referred to as marketable ears) were considered marketable and therefore harvested. Marketable ear number, green ear mass, and plant density were recorded. A subsample comprising ten randomly selected green ears was taken from each plot for measurements of ear length, filled ear length, kernel mass, and kernel moisture. Specifically, subsampled green ears were husked by hand and kernels were removed from the cob using an industry-grade hand-fed corn cutter (A&K Development, Eugene, OR). Husked ear mass and cob mass were recorded. Kernel mass was calculated as the difference between husked ear mass and cob mass. After that, recovery was calculated as the percentage of subsample green ear mass accounted by kernel mass. Fresh kernel samples (~100 g) were used to determine kernel moisture content gravimetrically at 55°C until dry.

In contract fields, sweet corn was hand harvested at three random locations outside the trial, as described above. These data were used to quantify current plant density and yield measurements at the current plant density. At the university research farm site, the current plant density was assigned 58,000 plants ha^-1^, the average plant density in the U.S. (Nick George, personal communication).

#### Economic analysis

Nearly all processing sweet corn in the U.S. is grown under contract [1], whereby the processor supplies seed of specific hybrids and decides on the planting density [16]. Growers of contract fields are paid (hereafter called ‘contract cost’) based on the mass of green ears (complete ears with husk leaves) the processor harvests from the field [16]. Processors quantify the performance of sweet corn based on recovery, cases of sweet corn produced per unit area (hereafter called ‘case production’), and gross profit margin. Each case contained 6.13 kg of kernels, moisture-corrected at 76 percent. Gross profit margin in the present work reflects the value of cases of sweet corn produced per hectare less seed cost and contract cost for green ear mass production. Economic analyses were based on U.S. dollars.

Hybrid seed cost was assumed to be $4.22 per 1,000 kernels. Contract cost ($ ha^-1^) was calculated using $82 as a fixed amount paid per unit metric ton of green ear mass harvested from the grower’s field. Similarly, gross returns ($ ha^-1^) to the processor were calculated as the product of total kernel case production (cases ha^-1^) and unit case price, fixed at $9.50. Finally, the processor’s gross profit margin ($ ha^-1^) was calculated by subtracting seed cost and the contract cost from gross returns. Economic estimates were verified by the Midwest Food Products Association (Nick George, personal communication).

#### Statistical analyses

Data were analyzed by fitting linear mixed effects models (LMEs) using the *nlme* package in RStudio [19, 20]. Individual models were fit to predict gross profit margin ($ ha^-1^), green ear mass (Mt ha^-1^) and, case production (cases ha^-1^), collectively referred to as ‘processor variables’. Each model was a second order polynomial mixed effects model with field-level random intercept and slope structure and plant density (plants ha^-1^) as the fixed effect. Field-level maximum values were calculated from the estimates of the best linear unbiased predictors (BLUPs) of random effects in the linear mixed effects model; the corresponding plant density (plants ha^-1^) was regarded as optimum plant density for maximizing crop response.

Additionally, linear mixed effects models with field as a random effect (random intercept and slopes) were constructed to study the fixed effect of plant density treatment on different response variables; namely, average ear length (cm), average filled ear length (cm), ear number per plant, green ear mass per plant (kg plant^-1^), kernel moisture (%) and recovery (%). Subsequent residual analysis was performed to check the normality assumption for all models. Means were compared using pairwise t-tests. For all analyses, significance was declared at α = 0.05.

## Results

### Linear mixed effects models

**Fig 1:**
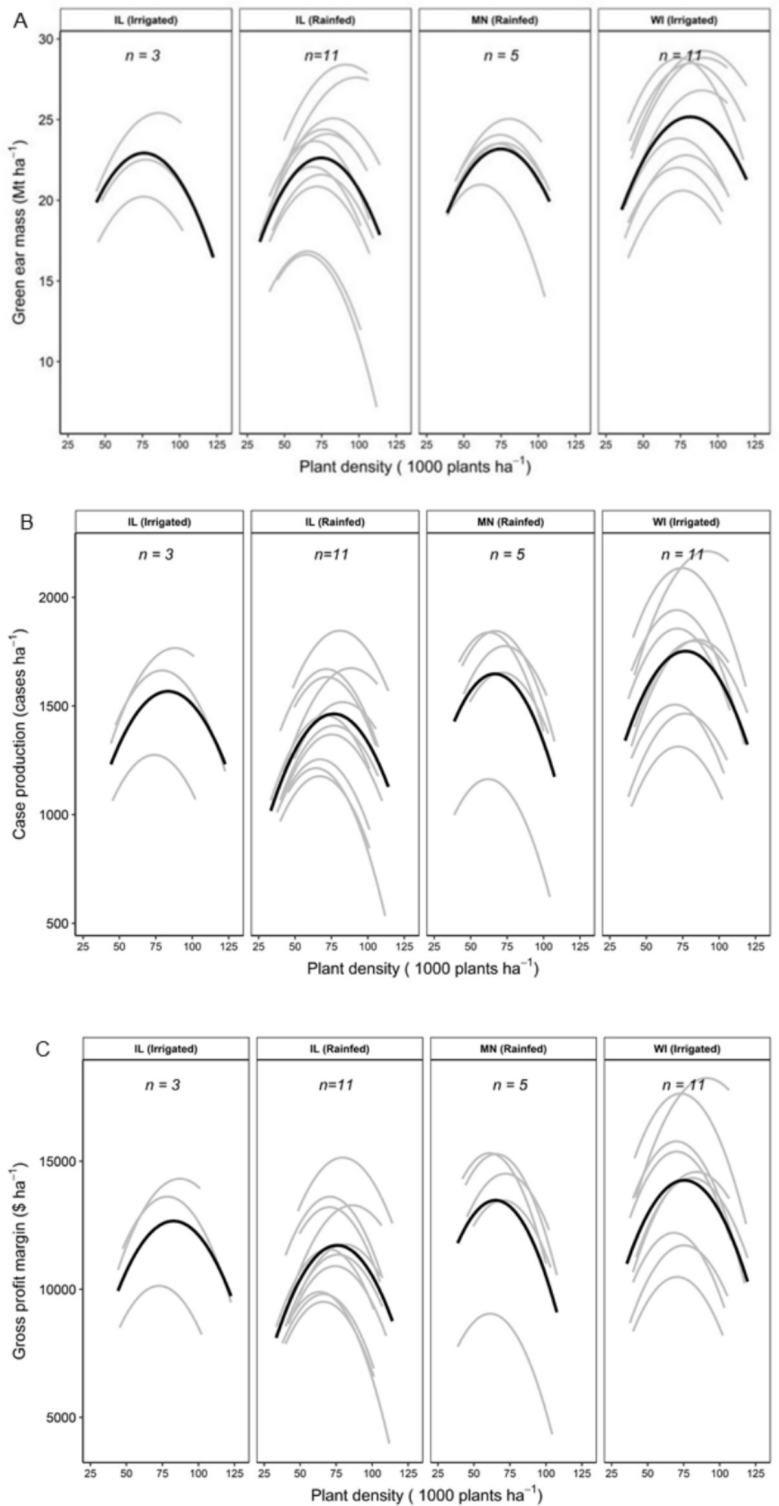
Linear mixed effects models of plant density effect of a crowding stress tolerant hybrid on (A) green ear mass (Mt ha^-1^), (B) case production (cases ha^-1^) and, (C) gross profit margin ($ ha^-1^) for four production areas. Thick black line is production area mean fixed effect. Grey lines are individual field relationships (best linear unbiased predictors, BLUPs), as estimated from the random effects structure.

Plant density affected all processor variables. In general, processor variables initially increased with plant density until reaching a peak at optimum plant density, and then began to decrease with higher plant density (Fig 1A–1C). Conditional R^2^_,_ the total amount of variation explained by the fixed and random effects using linear effects models [21], was 0.77, 0.74, and 0.73 for green ear mass, case production, and gross profit margin, respectively. Crop response to plant density varied within and across production areas (Fig 1A–1C).

#### Optimum plant densities

**Fig 2:**
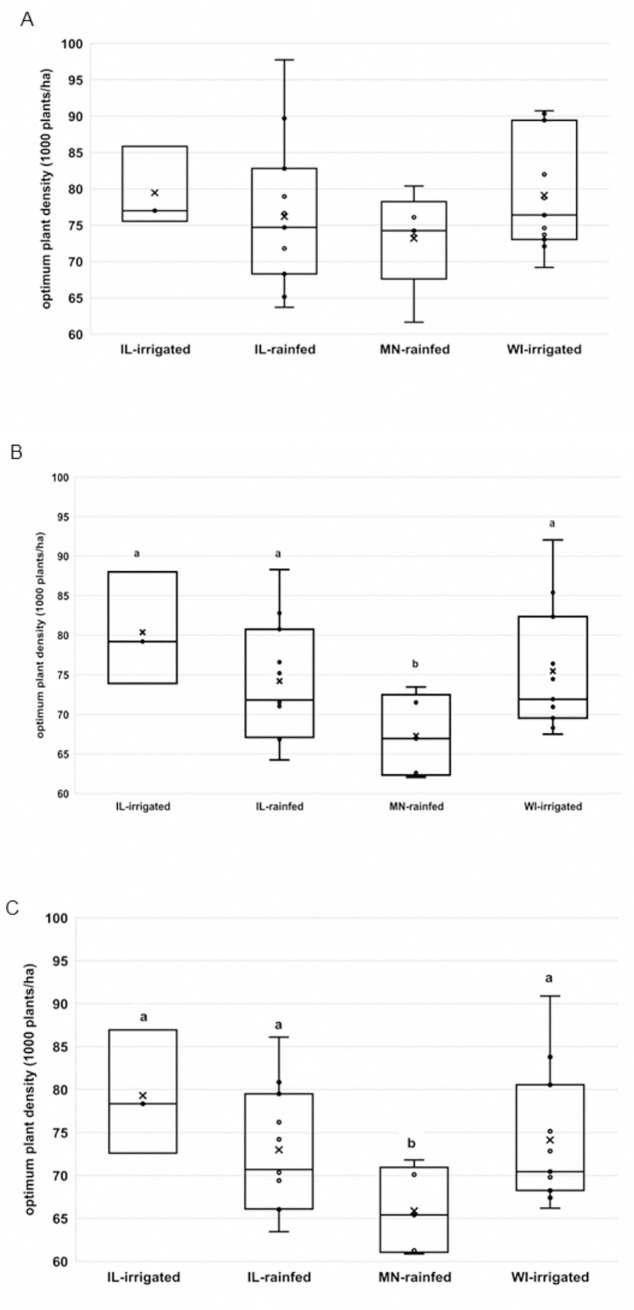
Box plots of optimum plant density distributions of a crowding stress tolerant hybrid in four production areas. Plant densities were optimized for (A) maximum green ear mass (Mt ha^-1^), (B) maximum case production (cases ha^-1^) (C) maximum gross profit margin ($ ha^-1^). Cross (x) sign represent means. Different letters denote significant differences in means at α = 0.05 based on pairwise t-tests.

Mean optimum plant density for maximum *green ear mass* ranged from 73,200 plants ha^-1^ in Minnesota-rainfed to 79,500 plants ha^-1^ in Illinois-irrigated (Fig 2A). However, no differences in optimum plant density for maximum green ear mass were observed among production areas.

Mean optimum plant density for maximum *case production* ranged from 65,900 plants ha^-1^ in Minnesota-rainfed to 79,300 plants ha^-1^ in Illinois-irrigated (Fig 2B). Mean optimum plant densities for Illinois-irrigated, Illinois-rainfed, and Wisconsin-irrigated production areas were greater than the Minnesota-rainfed production area.

Similarly, the mean optimum plant density for maximum *gross profit margin* ranged from 65,900 plants ha^-1^ in Minnesota-rainfed to 79,300 plants ha^-1^ in Illinois-irrigated (Fig 2C). Mean optimum plant density for Minnesota-rainfed was less than the mean optimum plant density at the other three production areas.

### Production gaps

Green ear mass yields at plant densities optimized for maximum *green ear mass* were higher than those observed at current plant densities across all four production areas (Table 2). Overall, increasing plant density from current plant densities (mean of 58,475 plants ha^-1^) to green ear mass-optimized plant densities (mean of 77,000 plants ha^-1^) added 1.18 Mt ha^-1^ of green ear mass.

**Table 2:** Comparison) of a crowding stress tolerant hybrid at current plant density and plant density optimized for maximum green ear mass (Mt ha^-1^), case production (cases ha^-1^) and gross profit margin ($ ha^-1^).

Yield measure	Production area	Current plant density	Optimum plant density	Difference in plant density	Current yield	Maximum yield	Difference in yield
Green ear mass		Plants ha^-1^			Mt ha^-1^		
	IL-irrigated	61,300	79,500	18,200*	21.74	22.77	1.03*
	IL-rainfed	58,400	76,200	17,800*	21.60	22.78	1.18*
	MN-rainfed	55,800	73,200	17,400*	22.33	23.44	1.11*
	WI-irrigated	58,400	79,100	20,700*	23.91	25.30	1.39*
**Overall mean**		**58,475**	**77,000**	18,525*	**22.4**	**23.57**	1.18*
Case production		Plants ha^-1^			Cases ha^-1^		
	IL-irrigated	61,300	79,300	18,000*	1,480	1,570	90*
	IL-rainfed	58,400	73,000	14,600*	1,400	1,475	75*
	MN-rainfed	55,800	65,900	10,100*	1,610	1,655	45
	WI-irrigated	58,400	74,100	15,700*	1,685	1,770	85*
**Overall mean**		**58,475**	**73,075**	14,600*	**1,544**	**1,618**	74*
Gross profit margin		Plants ha^-1^			$ ha^-1^		
	IL-irrigated	61,300	79,300	18,000*	12,000	12,700	700*
	IL-rainfed	58,400	73,000	14,600*	11,300	11,800	500*
	MN-rainfed	55,800	65,900	10,100*	13,200	13,500	300
	WI-irrigated	58,400	74,100	15,700*	13,800	14,400	600*
**Overall mean**		**58,475**	**73,075**	14,600*	**12,575**	**13,100**	525*

Asterisks represent significant differences between values at current and maximum at α = 0.05 based on pairwise t-tests.

Case production at plant densities optimized for maximum *case production* also were higher than those observed at current plant densities in Illinois-irrigated, Illinois-rainfed and Wisconsin-irrigated (Table 2). Minnesota-rainfed did not show any significant increase in case production at optimum plant density compared to current plant density. Overall, increasing plant density from current plant densities (mean of 58,475 plants ha^-1^) to case production-optimized plant densities (mean of 73,075 plants ha^-1^) added 74 cases ha^-1^.

Likewise, gross profit margin at plant densities optimized for maximum *gross profit margin* also were higher than those observed at current plant densities in Illinois-irrigated, Illinois-rainfed, and Wisconsin-irrigated (Table 2). Minnesota-rainfed did not show a significant increase in gross profit margin at optimum plant density compared to current plant density (Table 2). Overall, increasing plant densities from current plant densities (mean of 58,475 plants ha^-1^) to gross profit margin-optimized plant densities (mean of 73,075 plants ha^-1^) raised gross profit margin by $525 ha^-1^.

### Effect on yield and ear traits

Yield traits, specifically green ear mass and case production, were higher at plant densities optimized for maximum *gross profit margin* than those observed at current plant densities for all four production areas (Table 3). Overall, shifting from current to gross profit margin-optimized plant densities increased green ear mass and case production by 1.13 Mt ha^-1^ and 75 cases ha^-1^, respectively.

**Table 3:** Effect of a crowding stress tolerant hybrid on yield traits when shifting from current plant density to plant density optimized for maximum gross profit margin ($ ha^-1^) across different production areas.

Yield trait	Production area	Response at current plant density	Response at optimum plant density	Difference
Green ear mass		Mt ha^-1^		
	IL-irrigated	21.74	22.75	1.01*
	IL-rainfed	21.60	22.71	1.11*
	MN-rainfed	22.33	23.21	0.88*
	WI-irrigated	23.91	25.20	1.29*
**Overall mean**		**22.58**	**23.71**	1.13*
Case production		Cases ha^-1^		
	IL-irrigated	1,478	1,569	91*
	IL-rainfed	1,400	1,474	74*
	MN-rainfed	1,609	1,655	46
	WI-irrigated	1,684	1,770	86*
**Overall mean**		**1,547**	**1,622**	75*

Asterisks represent significant differences at α = 0.05 based on pairwise t-tests.

Ear traits, including ear number plant^-1^, ear mass plant^-1^, average ear length, and filled ear length were affected by plant density (Table 4). Overall, increasing from current plant density to gross profit margin-optimized plant density resulted in a subtle, yet statistically significant decline in ear number and ear mass by 0.08 ears plant^-1^ and 0.06 kg plant^-1^, respectively. Filled ear length showed a reduction (mean of 0.8 cm) compared to average ear length (mean of 0.5 cm) on shifting from current to gross profit margin-optimized plant density. Linear regression analysis showed ear number per plant decreased below 1.0 with increasing plant density (S1 Fig), indicating loss of marketable ears. Similarly, average ear length and filled ear length also decreased linearly with increasing plant density. More plants with unfilled ears at higher plant densities resulted in lower kernel mass, hence, lower case production and gross profit margin.

**Table 4:** Effect of a crowding stress tolerant hybrid on ear traits when shifting from current plant density to plant density optimized for maximum gross profit margin ($ ha^-1^) across different production areas.

Ear Trait	Production area	Response at current plant density	Response at optimum plant density	Difference
		Ears per plant		
Ear number per plant	IL-irrigated	1.07	0.86	-0.11*
	IL-rainfed	1.00	0.91	-0.08*
	MN-rainfed	1.00	0.96	-0.04*
	WI-irrigated	1.05	0.96	-0.09*
**Overall mean**		1.02	0.93	-0.08*
		Kg plant^-1^		
Ear mass per plant	IL-irrigated	0.36	0.29	-0.07*
	IL-rainfed	0.37	0.31	-0.06*
	MN-rainfed	0.40	0.36	-0.04*
	WI-irrigated	0.42	0.35	-0.07*
**Overall mean**		0.39	0.33	-0.06*
		cm		
Average ear length	IL-irrigated	19.4	19.0	-0.4*
	IL-rainfed	19.1	18.7	-0.4*
	MN-rainfed	19.9	19.4	-0.6*
	WI-irrigated	19.3	18.8	-0.5*
**Overall mean**		19.3	18.9	-0.5*
		cm		
Filled ear length	IL-irrigated	17.9	16.9	-1.0*
	IL-rainfed	17.6	16.9	-0.8*
	MN-rainfed	18.8	17.8	-1.0*
	WI-irrigated	18.3	17.5	-0.8*
**Overall mean**		18.1	17.3	-0.8*
		(%)		
Recovery	IL-irrigated	43.4	43.5	0.1
	IL-rainfed	42.2	42.2	0.0
	MN-rainfed	45.7	44.8	-0.9*
	WI-irrigated	45.4	45.3	-0.1
**Overall mean**		44.1	43.9	-0.2*
		(%)		
Kernel moisture	IL-irrigated	77.0	77.0	0.0
	IL-rainfed	77.3	77.3	0.0
	MN-rainfed	77.1	77.1	0.0
	WI-irrigated	77.5	77.6	0.1
**Overall mean**		77.3	77.4	0.1

Asterisks represent significant differences at α = 0.05 based on pairwise t-tests.

Overall, recovery declined by 0.2 percent by increasing plant densities from current plant density to gross profit margin-optimized plant density (Table 4). However, only Minnesota-rainfed showed a significant decrease in recovery (0.9 percent). Kernel moisture, an indicator of crop development, was unaffected as plant densities increased from current to gross profit margin-optimized plant densities across all four production areas.

## Discussion

The experimental approach used in this study enabled us to quantify the extent to which a modern CST hybrid can be used to immediately improve sweet corn production under a variety of growing conditions. Optimum plant densities for one of the several available CST hybrids were identified. By locating experimental sites at areas of high strategic importance for processing sweet corn production, the research took into account relevant spatial and temporal variability in which the crop is produced (e.g. soil types, planting dates, and local climate). Locating experiments in growers’ fields captured real diversity in crop management practices, including nutrient management and pest control. Previous research has shown that on-farm experiments accelerate adoption of new farm technology, compared to pilot projects, by demonstrating results under real-world conditions [22].

While some sweet corn germplasm has improved considerably for CST, plant densities have remained more or less constant for decades. Since the mid-1980s, recommended plant densities for processing sweet corn in Minnesota and Wisconsin have ranged from 45,000 to 54,300 plants ha^-1^ [23, 24]. Two decades later, surveys of growers’ fields showed slight change in plant density [16]. Our results demonstrate that, regardless of the processor variable, current plant densities are too low for CST sweet corn, by 10,100 to 18,200 plants ha^-1^. These results are consistent with previous studies in field corn that reported increased CST in modern hybrids allows using higher plant densities than their predecessors [3, 25, 26, 27].

Our results reveal that both processors and their contract growers will benefit from using gross profit margin-optimized plant densities of a CST hybrid like DMC 21–84. Overall green ear mass and case production increased by an average of 1.13 Mt ha^-1^ and 75 cases ha^-1^, respectively. Furthermore, these economic gains were achieved without altering crop management practices other than crop seeding rate, which was factored into the economic analysis. Moreover, there may be an environmental benefit to this research. Dry matter accumulation increases with plant density and promotes sequestration of nitrate as organically bound nitrogen [28, 29]. Although beyond the scope of this research, increasing plant density could reduce the amount of soil nitrate available for leaching following sweet corn harvest.

Sweet corn ear size and shape are important in the processing industry. For instance, slightly tapered ears are necessary to orient ears correctly using automated processing equipment [30]. Moreover, excessive variability in ear size and shape interferes with kernel cutting [30]. Our results show ear traits, at plant densities optimized for maximum gross profit margin, remain suitable for the mechanized processing. Moreover, [31] showed that ear number per plant is a relatively poor predictor of case production and gross profit margin in processing sweet corn (*ρ* = 0.679 and 0.661, respectively).

Kernel moisture and recovery showed minimal differences at current and optimum plant densities. Kernel moisture at optimized plant densities was within the desired range (74–78 percent) required for processing. This suggests that crop maturity was not delayed at optimum plant density. Also, sweet corn maturity is associated with kernel eating quality such as flavor and texture [32]. Mean recovery in 3 of 4 production areas was unaffected by optimum plant density. Total case production increased due to more ears per hectare at optimum plant densities than current plant densities. Minnesota-rainfed was the only production area showing decline in recovery at optimized plant density—by 0.9 percent—which may be due to an interaction between environmental factors and agronomic practices.

## Conclusion

Processing sweet corn yields have stagnated the last two decades in many, if not all, production regions of the world. Historic yield improvements in field corn are mostly the result of utilizing increased plant densities of CST germplasm. If field corn serves as any example, improving productivity of sweet corn will involve utilizing modern hybrids that maintain individual plant yield under higher plant densities than their predecessors. Earlier studies have documented CST germplasm is being under-planted in the major production region of the U.S. (at 56,000 plants ha^-1^), and this work shows the gross profit margin-optimized plant densities for CST processing sweet corn ranges from 65,900 to 79,300 plants ha^-1^, depending on production area. Optimum plant densities increased average yield to the grower by 1.13 Mt ha^-1^ and average gross profit margin to the processor by $525 ha^-1^ without negatively affecting ear traits important to processing. This study demonstrates that the processing sweet corn industry could benefit from CST germplasm, and planting such hybrids at densities that fully utilize their genetic potential.

## Supporting information

S1 Fig **Linear mixed effects models of plant density effects of a crowding stress tolerant hybrid on (A) ear number per plant, (B) average ear length (cm), and (C) filled ear length (cm) for four production areas.** Thick black line is production area mean fixed effect. Colored lines are individual field relationships (best linear unbiased predictors, BLUPs), as estimated from the random effects structure.
(PDF)

S1 File Raw data used for all analyses in the manuscript. (XLSX)
