# Supplementary figures and images for: Optimum plant density for crowding stress tolerant processing sweet corn
Source: PLoS One. 2019 Sep 26;14(9):e0223107. doi: 10.1371/journal.pone.0223107 (PMC6762085; doi:10.1371/journal.pone.0223107)

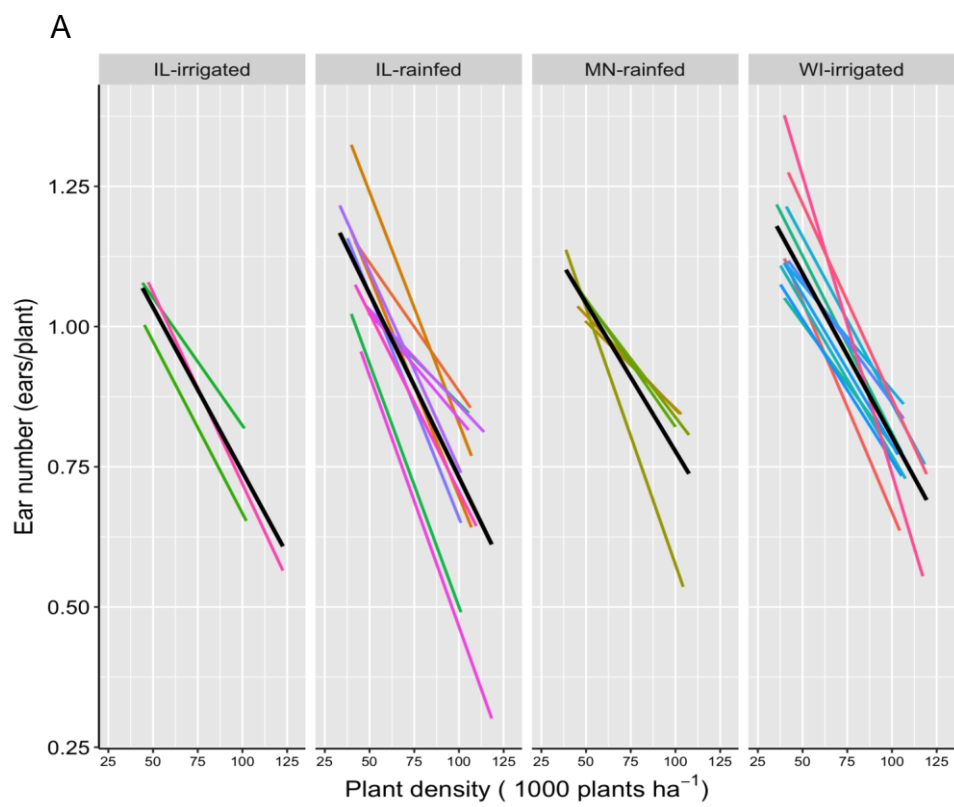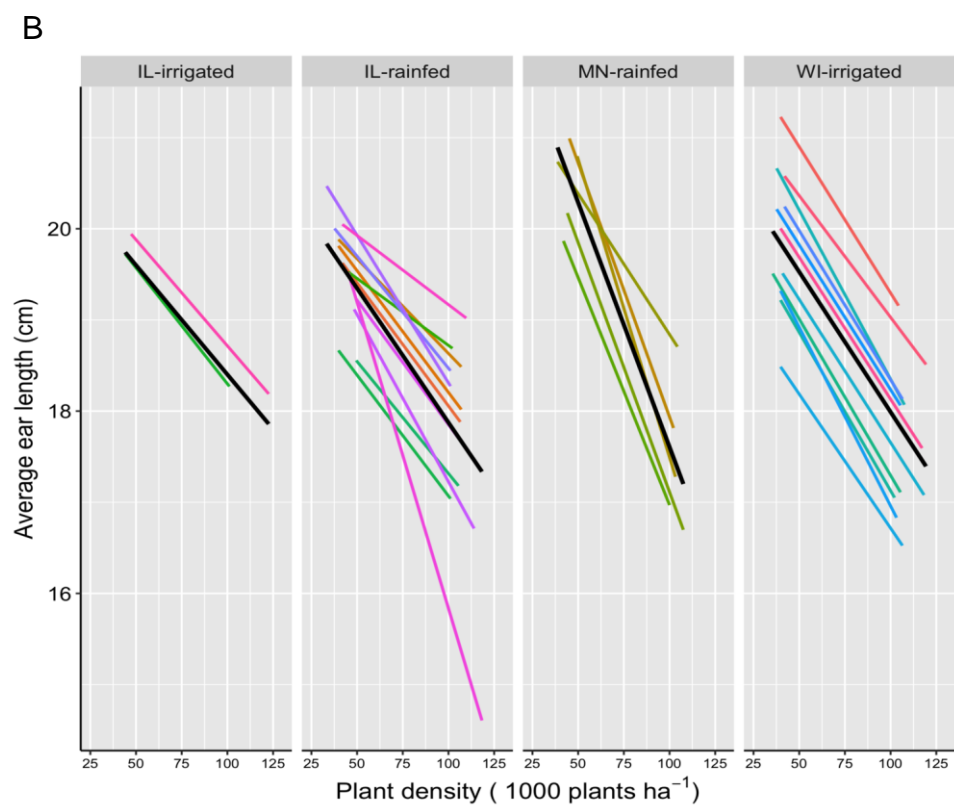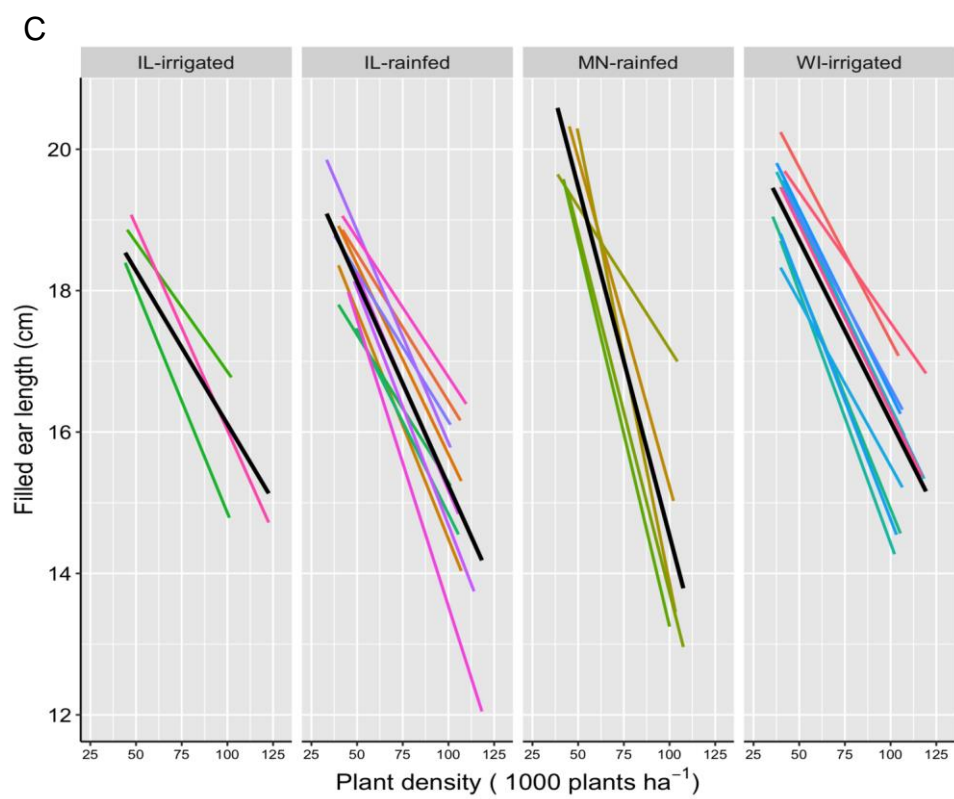

Supplement: S1 Fig — Linear mixed effects models of plant density effects of a crowding stress tolerant hybrid on (A) ear number per plant, (B) average ear length (cm), and (C) filled ear length (cm) for four production areas. Thick black line is production area mean fixed effect. Colored lines are individual field relationships (best linear unbiased predictors, BLUPs), as estimated from the random effects structure. (PDF) [file pone.0223107.s001.pdf]
